# Oxygen-sensing pathways and the pulmonary circulation

**DOI:** 10.1113/JP284591

**Published:** 2023-10-16

**Authors:** Mary E. Slingo

**Affiliations:** Department of Physiology, Anatomy and Genetics, University of Oxford, Oxford, UK

**Keywords:** hypoxia, hypoxia-inducible factor, hypoxic pulmonary vaso-constriction, iron, pulmonary circulation

## Abstract

The unique property of the pulmonary circulation to constrict in response to hypoxia, rather than dilate, brings advantages in both health and disease. Hypoxic pulmonary vaso-constriction (HPV) acts to optimise ventilation-perfusion matching – this is important clinically both in focal disease (such as pneumonia) and in one-lung ventilation during anaesthesia for thoracic surgery. However, during global hypoxia such as that encountered at high altitude, generalised pulmonary vasoconstriction can lead to pulmonary hypertension. There is now a growing body of evidence that links the hypoxia-inducible factor (HIF) pathway and pulmonary vascular tone – in both acute and chronic settings. Genetic and pharmacological alterations to all key components of this pathway (VHL – von Hippel–Lindau ubiquitin E3 ligase; PHD2 – prolyl hydroxylase domain protein 2; HIF1 and HIF2) have clear effects on the pulmonary circulation, particularly in hypoxia. Furthermore, knowledge of the molecular biology of the prolyl hydro-xylase enzymes has led to an extensive and ongoing body of research into the importance of iron in both HPV and pulmonary hypertension. This review will explore these relationships in more detail and discuss future avenues of research.

**Figure:**
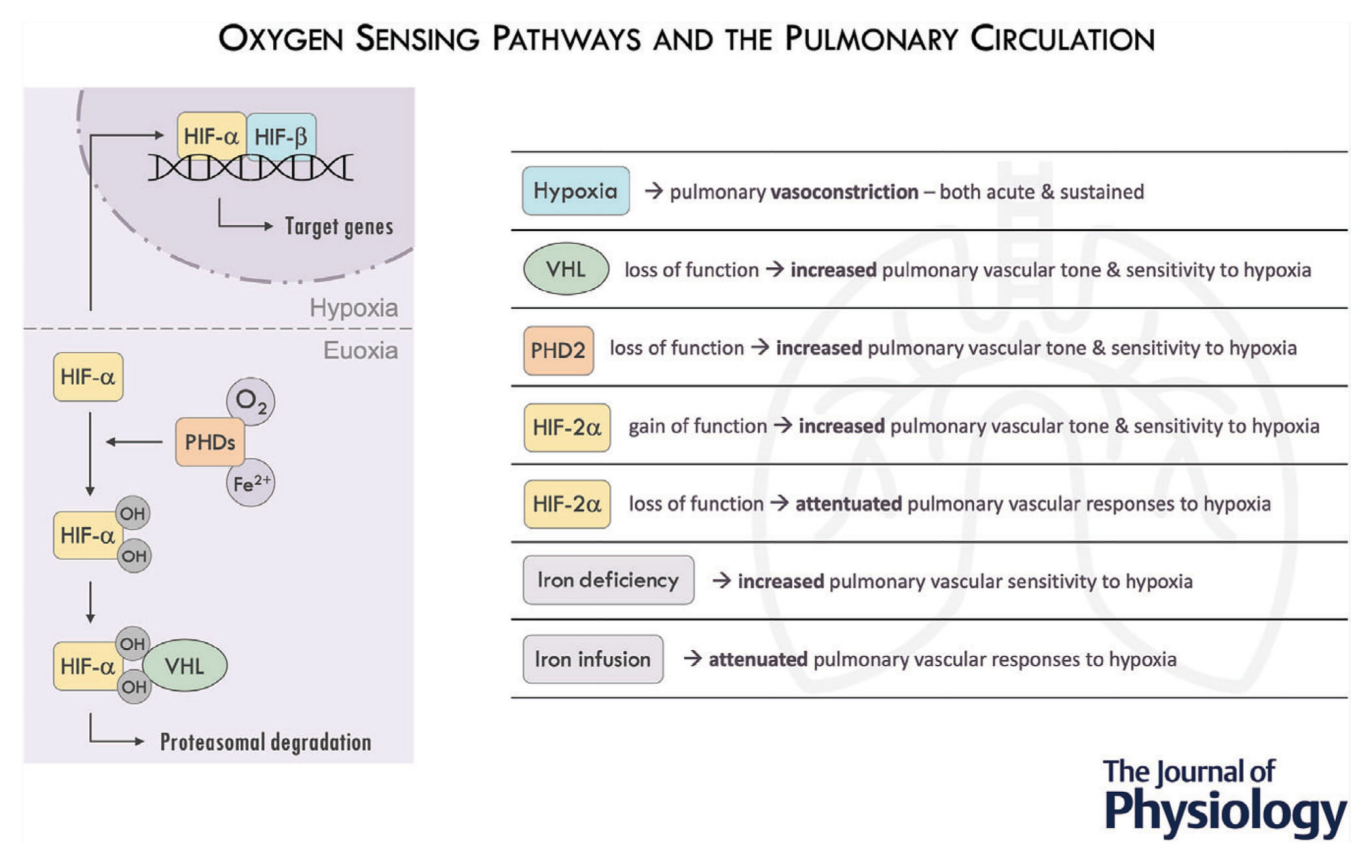
The heterodimeric transcription factor HIF (hypoxia-inducible factor) is thought to be responsible for the majority of adaptive transcriptional changes that take place in response to hypoxia. The stability of the HIF-*α* subunits is regulated by oxygen-dependent prolyl hydroxylation (by prolyl hydroxylase domain proteins, PHDs), which enables recognition by the von Hippel–Lindau (VHL) ubiquitin E3 ligase with subsequent degradation by the ubiquitin–proteasome pathway. In hypoxia, this process is impaired. There is now a growing body of evidence that links the HIF pathway and pulmonary vascular tone – in both acute and chronic settings. Genetic and pharmacological alterations to all key components of this pathway (VHL, PHD2, HIF1 and HIF2) have clear effects on the pulmonary circulation, particularly in hypoxia. Knowledge of the molecular biology of the prolyl hydroxylase enzymes has also led to an ongoing body of research into the importance of iron in both hypoxic pulmonary vasoconstriction and pulmonary hypertension.

The heterodimeric transcription factor HIF (hypoxia-inducible factor) is thought to be responsible for the majority of adaptive transcriptional changes that take place in response to hypoxia. The stability of the HIF-*α* subunits is regulated by oxygen-dependent prolyl hydroxylation (by prolyl hydroxylase domain proteins, PHDs), which enables recognition by the von Hippel–Lindau (VHL) ubiquitin E3 ligase with subsequent degradation by the ubiquitin–proteasome pathway. In hypoxia, this process is impaired. There is now a growing body of evidence that links the HIF pathway and pulmonary vascular tone – in both acute and chronic settings. Genetic and pharmacological alterations to all key components of this pathway (VHL, PHD2, HIF1 and HIF2) have clear effects on the pulmonary circulation, particularly in hypoxia. Knowledge of the molecular biology of the prolyl hydroxylase enzymes has also led to an ongoing body of research into the importance of iron in both hypoxic pulmonary vasoconstriction and pulmonary hypertension.

## Introduction

The unique property of the pulmonary vasculature to constrict in response to hypoxia and hypercapnia was first identified almost 80 years ago (Euler & Liljestrand, 1946) and is regarded as physiologically beneficial in both health and disease. Hypoxic pulmonary vasoconstriction (HPV) at a local level, in concert with hypercapnic vaso-constriction, optimises ventilation-perfusion matching – by decreasing the amount of blood flow that can occur through non-aerated and poorly ventilated lung regions, HPV assists in maintaining the arterial partial pressure of oxygen (PaO_2_). This is important clinically both in focal disease (such as pneumonia) and in one-lung ventilation during anaesthesia for thoracic surgery. However, the global hypoxia encountered at high altitude or in chronic lung disease can result in sustained generalised vaso-constriction that transforms the pulmonary circulation from a system of high capacity and distensibility to one of increased resistance and pressure.

**Figure 1:**
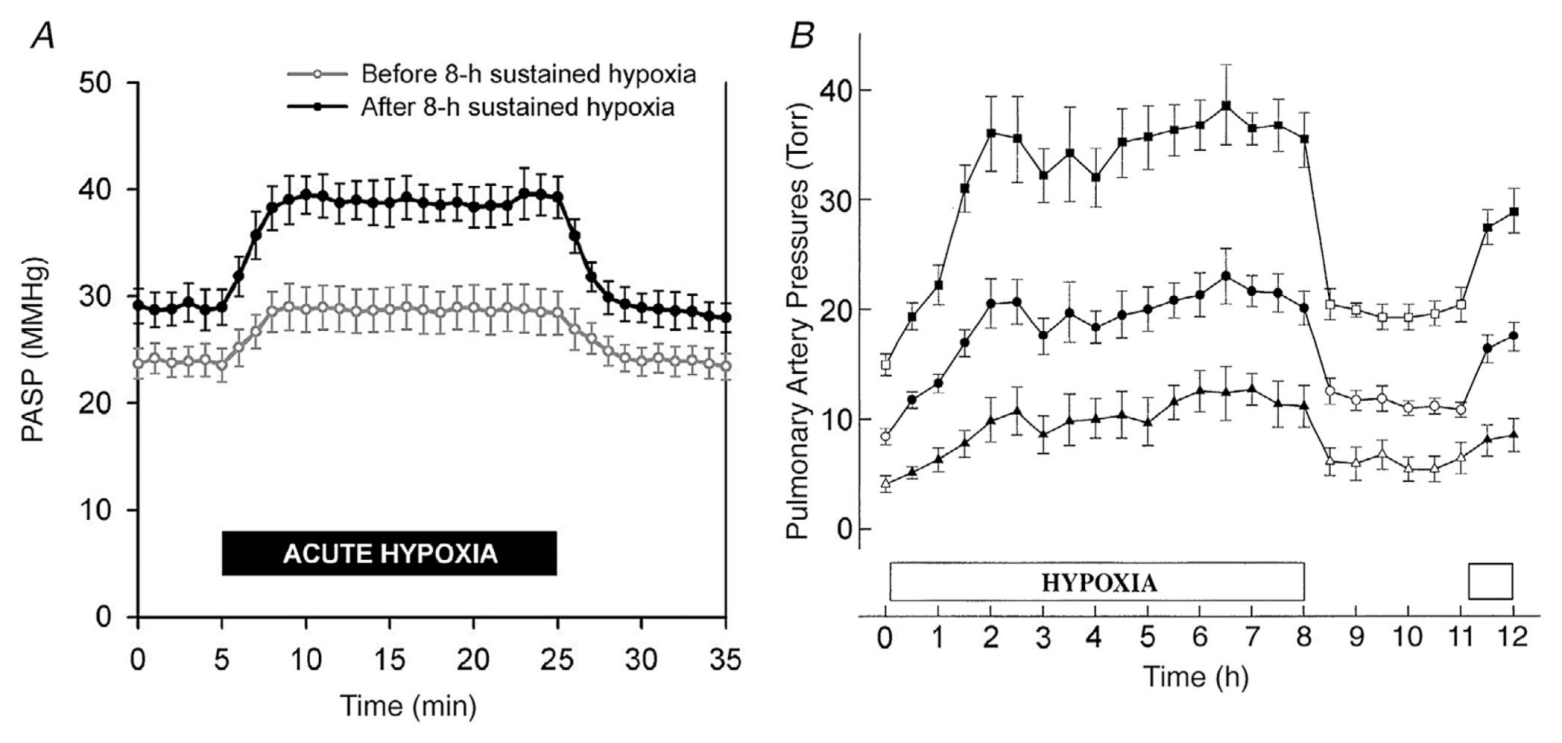
Pulmonary vascular responses to acute and sustained hypoxia *A*, pulmonary artery systolic pressure (PASP), measured using transthoracic echocardiography, in response to 20 min acute isocapnic hypoxia (50 mmHg), showing the characteristic rapid initial increase (open/grey circles). After a period of sustained hypoxia (closed/black circles), two features are illustrated – (1) baseline PASP is elevated; (2) there is a sensitisation of the acute response to a repeat hypoxic stimulus. From Smith et al. (2008), with permission. *B*, pulmonary artery pressure (systolic – squares; mean – circles; diastolic – triangles), measured using a pulmonary artery catheter, showing the time course over 8 h sustained hypoxia (50 mmHg) (black symbols). Pressures do not return to baseline on resumption of euoxia (open symbols), and a further hypoxic stimulus reveals sensitisation of the pulmonary vascular response. From Dorrington et al. (1997), with permission.

This transformation has several temporal components and differing degrees of reversibility (Fig. 1). The time course of the pulmonary responses to hypoxia is well-illustrated by both Dorrington et al. (1997) and Talbot et al. (2005) – there is an initial increase in vascular resistance within 60 min (most rapidly within the first 5 min), followed by a second phase comprising a progressive sustained rise over the next few hours. In addition, the pulmonary vascular resistance does not return immediately to normal levels after euoxia resumes, and there is a sensitisation of the acute response to a repeat hypoxic stimulus. If hypoxia continues, a remodelling of the pulmonary arterial vasculature takes place, involving structural changes that result in increased vessel stiffness, decreased luminal diameter, and overall increased resistance to blood flow.

The mechanism(s) underlying the initial rapid phase of HPV are not yet fully elucidated, but certainly involve constriction of pulmonary artery smooth muscle cells (PASMCs) in response to an increase in intra-cellular calcium. How a reduction in oxygen causes this constriction remains an area of active research and has been reviewed in detail elsewhere (Sylvester et al., 2012). In contrast, the pulmonary vascular changes in response to sustained hypoxia (both the remodelling and the increased responsiveness) suggest that changes in gene expression and new protein synthesis are involved. A myriad of players have been identified and reviewed in detail (Pullamsetti et al., 2020; Shimoda, 2020), and it is unsurprisingly the hypoxia-inducible factor (HIF) pathway that has received particular attention – there is a growing body of evidence that links all major components of this signalling cascade with pulmonary vascular tone.

## The oxygen-sensing (HIF) pathway

All animal life requires molecular oxygen as the terminal electron acceptor in aerobic energy production, and thus animals have evolved sophisticated mechanisms to detect and react to changes in oxygen availability. Central to this is oxygen-dependent post-translational modification of a conserved transcription factor termed the ‘hypoxia-inducible factor’ (HIF).

HIF is a heterodimer, comprising an oxygen-regulated *α*-subunit and a ubiquitous *β*-subunit (also known as aryl hydrocarbon receptor nuclear translocator). The stability of the HIF-*α* subunits is regulated by oxygen-dependent prolyl hydroxylation (by prolyl hydroxylase domain proteins, PHDs), which enables recognition by the von Hippel–Lindau (VHL) ubiquitin E3 ligase with subsequent degradation by the ubiquitin–proteasome pathway (Semenza, 2020). In addition to dioxygen and 2-oxoglutarate as substrates, the PHD enzymes also require ferrous iron at their catalytic centres.

**Figure 2:**
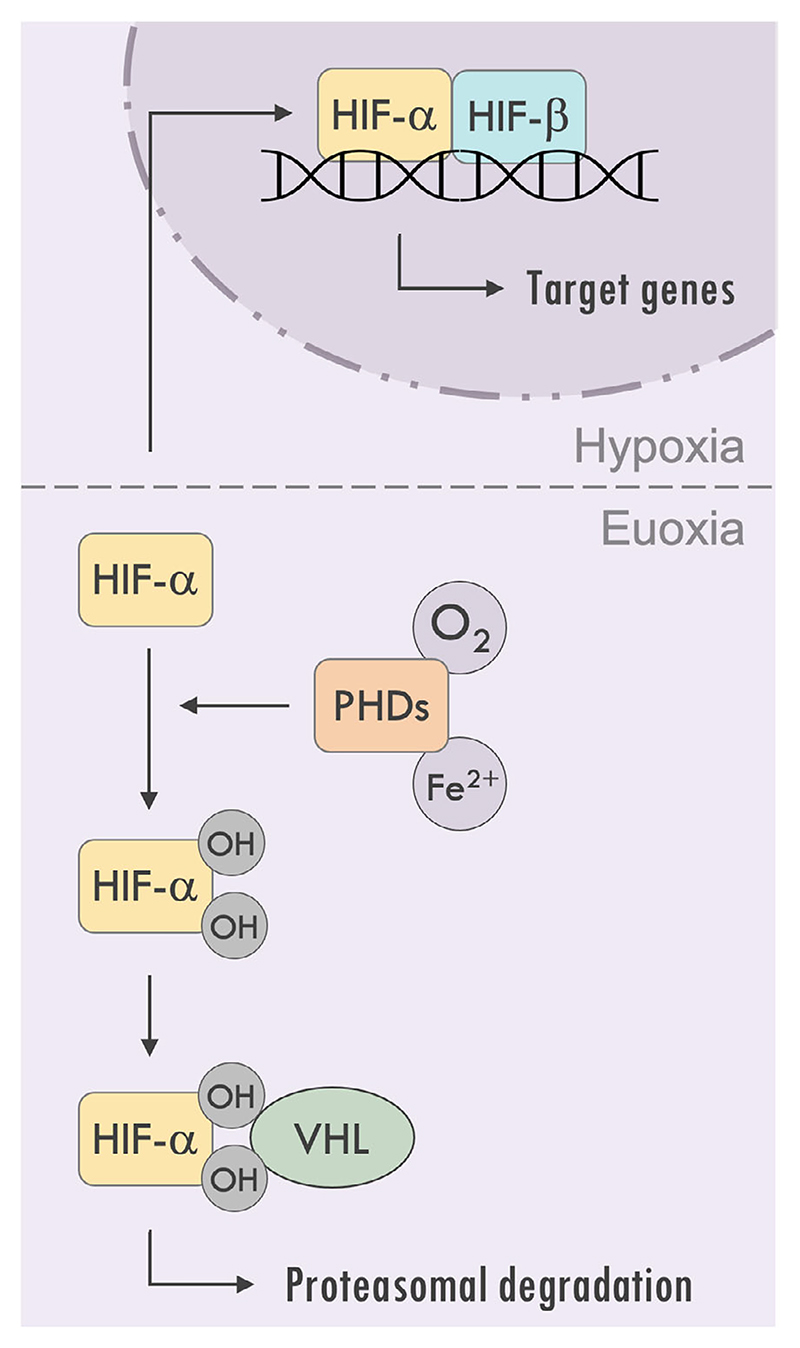
In euoxia, hypoxia-inducible factor (HIF)-*α* is rapidly hydroxylated by PHD enzymes and targeted for degradation by VHL When oxygen becomes limiting, HIF-*α* is able to accumulate and bind with HIF-*β* in the nucleus to function as a transcription factor. Fe^2+^, ferrous iron; O_2_, oxygen; OH, hydroxyl group; PHD, prolyl hydroxylase domain enzyme; VHL, von Hippel–Lindau protein.

During hypoxia, and potentially also when iron is limiting, the enzymatic process is inhibited and therefore HIF-*α* is able to accumulate. This can result in the transcription of many hundred genes involved in diverse processes such as angiogenesis, erythropoiesis and cellular metabolism. Thus, the hydroxylation step provides a mechanism that directly transduces changes in oxygen availability to altered gene expression. In humans, there are two key paralogues of HIF-*α* with slightly different roles (HIF-1*α* and HIF-2*α*). Their regulation differs, and the balance between them may be important for controlling temporal- and tissue-specific differences in responses to hypoxia. A third paralogue, HIF-3*α*, has been identified but is the least well studied. The main HIF pathway is summarised in Fig. 2.

In mammals, this HIF pathway is widespread and thought to be responsible for the majority of adaptive transcriptional changes that take place in response to hypoxia. Recently, additional systems of enzymatic protein oxidation (with subsequent protein degradation) have been identified – remarkably, one of these has been shown to be equivalent to the predominant system found in plants (Hammarlund et al., 2020; Holdsworth & Gibbs, 2020; Tian et al., 2023).

## The HIF pathway and the pulmonary circulation

**Figure 3:**
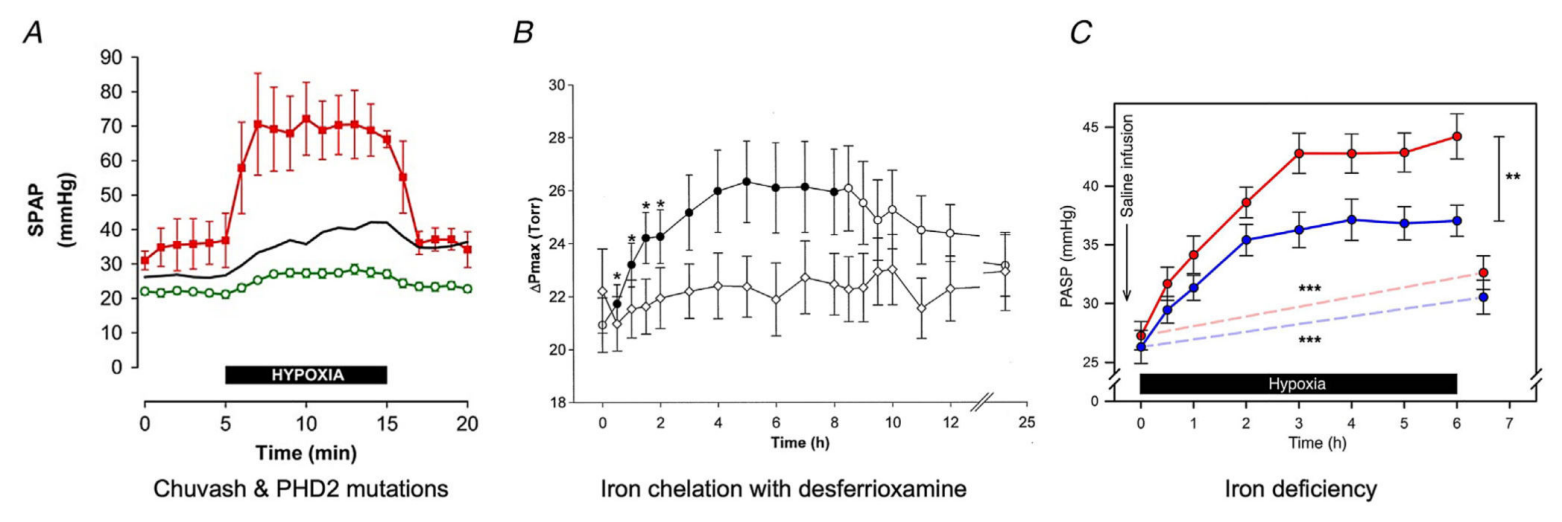
A variety of genetic, pharmacological and clinical conditions result in upregulation of the hypoxia-inducible factor (HIF) pathway with accompanied increased pulmonary vascular responses to hypoxia *A*, Chuvash polycythaemia patients (red line and markers) have significantly elevated baseline systolic pulmonary artery pressure (SPAP) and a marked sensitivity of the pulmonary vascular response to acute isocapnic hypoxia (50 mmHg) compared with controls (green). An individual with a PHD2 gain-of-function mutation (black line) has an intermediate phenotype. From Talbot et al. (2017), with permission. *B*, iron chelation using a desferrioxamine infusion (closed/black circles) induces a significant and sustained increase in ΔPmax in euoxia compared with controls (open/white). From Balanos et al. (2002), with permission. *C*, the increase in pulmonary artery systolic pressure (PASP) during hypoxia is significantly greater in individuals with iron deficiency (red line and markers) compared with controls (blue line and markers). From Frise et al. (2016), with permission. PHD, prolyl hydroxylase domain enzyme.

**Figure 4:**
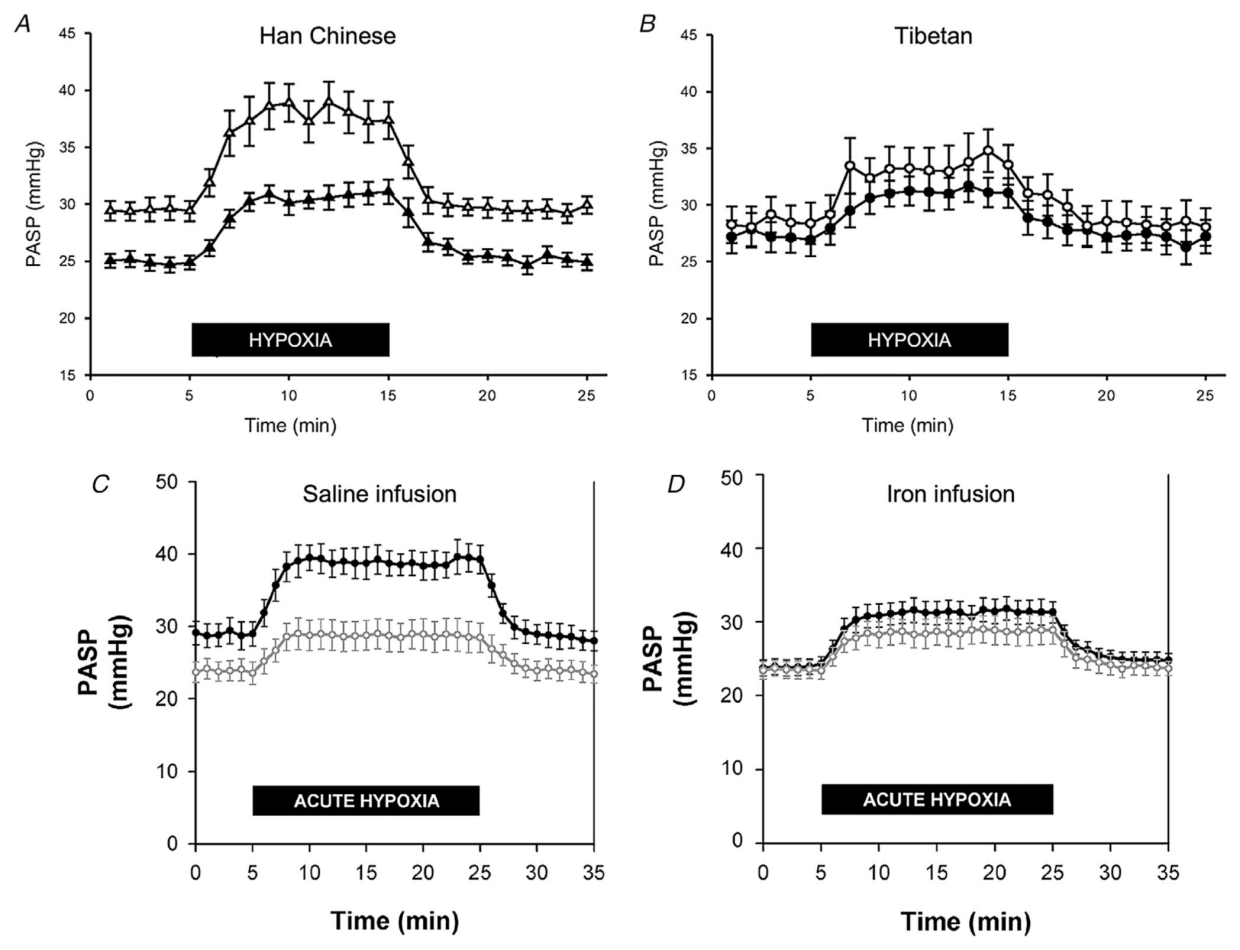
A hyporesponsive hypoxia-inducible factor (HIF) system is associated with attenuated pulmonary vascular responses to acute and sustained hypoxia *A* and *B*, compared with Han Chinese controls, Tibetans living at sea level have blunted pulmonary vascular responses to acute isocapnic hypoxia (50 mmHg; black markers). They also have both an attenuated response to 8 h sustained hypoxia (white markers: minimal elevation in baseline pulmonary artery systolic pressure (PASP) occurs), and reduced sensitivity of the pulmonary vascular response to a second acute hypoxia exposure (white markers: compared with Han Chinese, the increase in PASP during acute hypoxia is markedly attenuated). From Petousi et al. (2014), with permission. *C* and *D*, a very similar pattern is seen with intravenous iron supplementation. The normal responses to acute hypoxia before (white markers) and after (black markers) 8 h sustained hypoxia with a prior saline (control) infusion are shown in panel *C*. In contrast, if an iron infusion is administered prior to sustained hypoxia the pulmonary vascular responses are substantially diminished (*D*). From Smith et al. (2008), with permission.

The first direct evidence linking the HIF pathway and the pulmonary circulation comes from the finding that heterozygous deletion of HIF-1*α* in mice attenuates the development of hypoxic pulmonary hypertension (Yu et al., 1999), and those with heterozygous deletion of HIF-2*α* are completely protected (Brusselmans et al., 2003). By considering the molecular biology of the oxygen-sensing pathway, one could anticipate that other genetic or pharmacological alterations to its key components (VHL, PHDs, HIFs) may have an effect on pulmonary vascular tone, and indeed this is the case (summarised in Figs 3 and 4).

### Von Hippel–Lindau (VHL)

The archetypal genetic disease of oxygen-sensing is Chuvash polycythaemia (CP), a rare autosomal recessive disorder caused by a germline point mutation in VHL. This mutation results in diminished binding affinity of the VHL protein for hydroxylated HIF-*α*, thus increasing the expression of HIF target genes under euoxic conditions (Ang et al., 2002).

Patients and mice with CP develop striking physiology which includes marked polycythaemia, increased ventilatory sensitivity to hypoxia, and altered skeletal muscle metabolism and energetics (Formenti et al., 2010; Slingo et al., 2014; Smith et al., 2006). Of note, affected individuals have elevated resting pulmonary artery systolic pressure (PASP) and an exaggerated pulmonary vasoconstrictive response to both mild and moderate hypoxia (Smith et al., 2006). Pulmonary hypertension, with accompanying changes in the right ventricle, has also been demonstrated in the mouse model (Hickey et al., 2010; Slingo et al., 2016).

Since the identification of the CP mutation, other polycythaemic individuals with different missense mutations in both VHL alleles have been shown to have pulmonary hypertension (Bond et al., 2011; Botros et al., 2017; Caravita et al., 2016; Chomette et al., 2022; Sarangi et al., 2014) and increased pulmonary vascular responses to hypoxia (Perrotta et al., 2020). In addition, there are carriers of other homozygous VHL mutations with polycythaemia in whom the pulmonary vasculature has not been investigated (Pastore et al., 2003; Tomasic et al., 2013).

In contrast, individuals heterozygous for the VHL cancer syndrome mutation (which requires a ‘second hit’ mutation/inactivation in the tumour cells) do not develop polycythaemia (as a result of the mutation) or pulmonary hypertension (Formenti et al., 2011). One explanation for this is that the homozygous CP genotype is associated with a reduced level of systemic VHL activity that is sufficient to prevent tumorigenesis but not fully able to properly regulate erythropoiesis. Whereas the presence of a single VHL syndrome allele results in adequate systemic VHL activity to prevent polycythaemia, when the wild-type allele is lost the residual cellular VHL activity is unable to prevent tumorigenesis (Semenza, 2020).

### PHD2

As the previous section demonstrates, VHL loss-of-function is a well-established upregulator of the HIF pathway that can lead to polycythaemia and pulmonary hypertension in euoxia. One would expect impaired action of the prolyl hydroxylase enzymes for HIF-*α* to have a similar effect, and indeed this is the case.

PHD2 in particular appears to have an important role – multiple groups have demonstrated that mice with endothelial cell-specific PHD2 deletion develop spontaneous pulmonary hypertension (Dai et al., 2016; Elamaa et al., 2022; Kapitsinou et al., 2016; Tang et al., 2018; Wang et al., 2016). This is in contrast with induced generic smooth muscle cell loss of PHD2 in mice, which does not augment right ventricular systolic pressure (RVSP) unless hypoxic pulmonary hypertension is established first (Chen et al., 2016). This study has some limitations due to the generic loss of PHD2 in all smooth muscle cells. More recently, a novel post-natal PHD2 deletion in only PASMCs has resulted in increased RVSP but no remodelling or other histopathological changes (Elamaa et al., 2022).

Whilst VHL mutations are a relatively common cause of HIF pathway diseases, PHD2 mutations in humans are seen less often. Nevertheless, one individual with heterozygous PHD2 loss has been studied – whilst ventilatory responses to moderate hypoxia were similar to those seen in patients with CP, the pulmonary vascular response was less marked. The baseline PASP was only mildly elevated, and during hypoxia the magnitude of rise in PASP appeared to be intermediate between the normal response and the exaggerated response seen in CP patients (Talbot et al., 2017). One potential explanation for this discrepancy is the differing degree of iron deficiency between these two populations (as discussed later).

In addition to genetic studies, pharmacological manipulation of the prolyl hydroxylase enzymes is now possible. Indeed, roxadustat (FG-4592, FibroGen) is a PHD inhibitor that is now licensed in many countries for the treatment of anaemia associated with chronic kidney disease (Chen et al., 2019a, 2019b). It is interesting to note that there has been one case report of associated pulmonary hypertension (Cygulska et al., 2019). Finally, whilst the PHD2 activator R59949 was unable to reverse the elevated RVSP seen in wild-type mice exposed to hypoxia, it did decrease the hypoxia-induced pulmonary arterial wall thickening (Chen et al., 2016).

Taken together, these studies in both mice and humans demonstrate the important role that the prolyl hydro-xylase enzymes, and PHD2 in particular, play within the pulmonary circulation.

### HIF-2*α*

Alterations in VHL and PHD function ultimately affect the stabilisation of HIF-*α* in euoxia. It is therefore unsurprising that genetic and pharmacological manipulation of HIF itself would result in changes within the pulmonary circulation. Of the three isoforms, whilst HIF-1*α* clearly has a role to play (Pullamsetti et al., 2020), it appears that HIF-2*α* is of particular importance. In contrast with VHL and PHD, both gain- and loss-of-function perturbations of HIF-2*α* have been described, resulting in either activation or inhibition of the oxygen-sensing pathway with consequent contrasting physiology.

Germline heterozygous gain-of-function mutations in HIF-2*α* are known to result in polycythaemia, and a small number of these patients have been studied to investigate their pulmonary vascular responses to hypoxia. As would be predicted, these individuals have both significantly higher PASP at baseline, and a significantly greater increase in PASP in response to moderate hypoxia (Formenti et al., 2011). One of these mutations has been further characterised in a mouse model, confirming that the erythrocytosis and pulmonary hypertension are present with a high degree of penetrance and in a mutation dose-dependent manner (Tan et al., 2013).

Further evidence of the importance of HIF-2*α* in pulmonary vascular tone is provided by the bovine condition known as Brisket disease, in which lowland cattle develop severe pulmonary hypertension during their summer migration to high-altitude grazing in Colorado. A newly identified, most likely gain-of-function, double variant in EPAS1 (HIF-2*α*) has been shown to be highly associated with this high-altitude pulmonary hypertension (Newman et al., 2015).

In contrast, mice with global heterozygous deletion of HIF-2*α* are completely protected from hypoxic pulmonary hypertension (Brusselmans et al., 2003), as are those with HIF-2*α* deleted specifically in the pulmonary endothelium (Cowburn et al., 2016; Tang et al., 2018). When mice with either VHL or PHD2 mutations develop pulmonary hypertension, it is HIF-2*α* (via concomitant deletion) that has been shown to be the critical downstream effector (Dai et al., 2018; Hickey et al., 2010; Kapitsinou et al., 2016).

Whilst initially technically challenging to develop, pharmacological inhibitors of HIF-2*α* are now showing promise as anti-cancer treatments. Intriguingly, they have also been shown to have potential in treating different animal models of pulmonary hypertension. C76, for example, is a selective HIF-2*α* translation inhibitor which significantly attenuates the increased RVSP seen in Sugen-hypoxia rats, in monocrotaline-exposed rats and in PHD2 knockout mice (Dai et al., 2018). In addition, knockdown of HIF-2*α* by antisense-oligonucleotides significantly attenuates hypoxia-induced pulmonary hypertension in adult mice (Hu et al., 2019). However, one disadvantage of both C76 and antisense-oligonucleotides is that they can only be administered by injection – oral HIF-2*α* inhibitors have now been developed and indeed one is licensed already for the treatment of renal cell carcinoma in VHL disease (Jonasch et al., 2021). Of note, belzutifan (PT2977/MK6482) has now been shown to significantly reduce the pulmonary hypertension, echocardiographic changes and polycythaemia seen in mice with the Chuvash mutation (Ghosh et al., 2021). Furthermore, an alternative oral inhibitor, PT2567, has been shown to attenuate the pulmonary hypertension seen in hypoxia (Hu et al., 2019), and in both Sugen-hypoxia and monocrotaline models (Macias et al., 2021). Finally, a third inhibitor, PT2385, is effective in both hypoxia (Wang et al., 2021) and Sugen-hypoxia animal models (Zheng et al., 2022). It is likely to be only a matter of time before trials in human participants are underway.

### Human adaptation at high altitude

Humans have inhabited low-oxygen high-altitude environments for many hundreds of generations, most notably in the Tibetan, Andean and Ethiopian highlands. Hypoxia is a strong driver of evolutionary adaptation in many species (Hall et al., 2020; Pamenter et al., 2020), and these populations exhibit unique physiology and striking genetic signatures. The challenge of linking genotype with phenotype is considerable, despite the identification of multiple genomic signatures converging on the HIF pathway (Hall et al., 2020; Pamenter et al., 2020). One key physiological trait exhibited by these populations is that, on average, Tibetan and Ethiopian highlanders tend to have lower haemoglobin concentrations relative either to acclimatised lowlanders, or to Andeans at high altitude. Relationships between this haemoglobin phenotype and candidate genes exhibiting signals of selection, including those within the HIF pathway (EGLN1/PHD2 and EPAS1/HIF-2*α*), have now been demonstrated (Beall et al., 2010; Simonson et al., 2010; Yi et al., 2010). Remarkably, the EPAS1/HIF-2*α* haplotype associated with altitude adaptation in Tibetans has been shown to be most likely the product of introgression (gene flow) from archaic Denisovan (an extinct hominid) or Denisovan-related populations (Huerta-Sanchez et al., 2014).

As well as having an attenuated erythropoietic system, Tibetans also have strikingly blunted pulmonary vascular responses to hypoxia, both at altitude and at sea level (Groves et al., 1993; Petousi et al., 2014). Furthermore, whilst not reaching statistical significance, there is evidence of a general tendency for the ‘Tibetan’ EPAS1/HIF-2*α* allele to be associated with attenuated hypoxic responses across most physiological parameters tested (Petousi et al., 2014). Larger-scale studies would be required to confirm connections between genotype and phenotype, but it remains clear that prolonged habitation in low-oxygen environments has driven adaptive changes across multiple species.

## Iron and the pulmonary circulation

The absolute requirement of the prolyl hydroxylase enzymes for dioxygen creates a surprisingly simple mechanism to directly transduce changes in oxygen availability to altered gene expression. Similarly, the requirement for ferrous iron at the catalytic centre raises the possibility that PHD activity could also be sensitive to intracellular iron availability (Schofield & Ratcliffe, 2004).

Indeed, iron chelation with desferrioxamine (DFO) *in vitro* induces HIF-1 activity and erythropoietin mRNA expression with a time course that is similar to hypoxia (Wang & Semenza, 1993). Conversely, supplementation with iron suppresses HIF-1*α* levels in a manner dependent on a functional PHD system (Knowles et al., 2003). Furthermore, it has been shown that systemic iron deficiency in rats results in increased lung expression of both HIF-1*α* and HIF-2*α*, together with pulmonary hypertension and pulmonary vascular remodelling (Cotroneo et al., 2015). Restricting iron deficiency to just the PASMCs in mice leads to upregulation of the vaso-constrictor endothelin-1 (a well-established HIF target gene), as well as spontaneous pulmonary hypertension that is partially reversed by treatment with intravenous iron (Lakhal-Littleton et al., 2019).

Translating this *in vitro* and animal work into meaningful *in vivo* human physiology and clinical relevance has been the subject of intensive study over the past few decades. Early on, iron chelation by an infusion of DFO in healthy volunteers was shown to elevate PASP in euoxia over a similar time course to that seen with HPV (Balanos et al., 2002), as well as significantly augmenting the response seen in acute hypoxia (Smith et al., 2008). In addition, multi-day venesection of individuals with chronic mountain sickness (who already have elevated haemoglobin and PASP) results in progressive iron deficiency and an overall increase in PASP by approximately 25% (Smith et al., 2009).

In contrast, infusion of iron prior to sustained hypoxia substantially reduces both the increased baseline PASP and the enhanced sensitivity of the pulmonary vasculature to a further acute hypoxic stimulus (Smith et al., 2008). The effect of iron supplementation is notably restricted to the second phase of HPV, when HIF-mediated gene transcription is presumed to be acting – the rapid initial reflex response is preserved (Talbot et al., 2014). Beyond the laboratory setting, iron infusion after ascent to high altitude has been shown to reverse approximately 40% of the increased PASP resulting from the sustained hypobaric hypoxia (Smith et al., 2009). These effects of supra-physiological iron supplementation are long-lived – a single dose of intravenous iron continues to attenuate HPV at clinically relevant levels for at least six weeks (Bart et al., 2016).

Acute manipulation of iron status has provided valuable insights into pulmonary physiology in hypoxia. However, the rapid and substantial fluxes in iron availability resulting from both infusion and chelation are very different to the chronic, insidious nature of the iron deficiency seen in clinical practice. Nevertheless, whilst PASP in euoxia does not differ between iron-deficient individuals and iron-replete matched controls, the rise in PASP in response to sustained hypoxia has been shown to be significantly exaggerated – this is reversed by subsequent iron administration (Frise et al., 2016). Iron deficiency is a very common nutritional disorder, and is particularly prevalent in individuals with pulmonary hypertension (although debate surrounding the most appropriate definition of ‘iron deficiency’ in this context continues) (Cleland et al., 2023; Martens et al., 2023). Building on the evidence described above, as well as small clinical studies, a double-blinded, randomised, placebo-controlled crossover study was conducted that sought to explore the effects of intravenous iron supplementation in patients with pulmonary arterial hypertension (Howard et al., 2021). Whilst this trial showed no association between iron administration and either exercise capacity or cardiopulmonary haemodynamics, several limitations have been discussed and the importance of iron in the pulmonary circulation remains an open avenue of research (Krasinkiewicz & Lahm, 2021).

## Conclusions

The high-capacity low-pressure pulmonary circulation is a dynamic system able to respond to a wide variety of stimuli, particularly hypoxia. The discovery of the oxygen-sensing HIF pathway has led to an expansion in our understanding of how changes in the pulmonary vasculature are orchestrated. Genetic and pharmacological alterations to all key components of this pathway (VHL, PHDs and HIFs) have clear effects on pulmonary vascular tone, particularly in hypoxia. In addition, knowledge of the molecular biology of the prolyl hydroxylase enzymes has led to an extensive and ongoing body of research into the importance of iron in both HPV and pulmonary hypertension. Finally, with increasing numbers of HIF pathway modulators entering clinical practice, understanding their long-term effects on the pulmonary circulation will be an important focus of future research.
